# Brain and the whole-body bone imaging appearances in Menkes disease: a case report and literature review

**DOI:** 10.1186/s12887-024-04885-x

**Published:** 2024-06-26

**Authors:** Juncheng Zhu, Yi Liao, Xuesheng Li, Fenglin Jia, Xinmao Ma, Haibo Qu

**Affiliations:** 1 Department of Radiology, West China Second University Hospital of Sichuan University, Chengdu, 610041 Sichuan Province People’s Republic of China; 2 Department of Radiology, Chengdu Seventh People’s Hospital (Affiliated Cancer Hospital of Chengdu Medical College), Chengdu, 610213 Sichuan Province People’s Republic of China; 3 Key Laboratory of Birth Defects and Related Diseases of Women and Children (Sichuan University), West China Second University Hospital, Ministry of Education, Sichuan University, Chengdu, 610041 Sichuan Province People’s Republic of China

**Keywords:** Menkes disease, Copper metabolism disorder, Brain, Bone, Image

## Abstract

**Background:**

Menkes disease (MD) is a rare, inherited, multisystemic copper metabolism disorder. Classical Menkes disease is characterized by low serum copper and ceruloplasmin concentrations, leading to multiple abnormalities in the whole-body, especially in connective tissue and central nervous system. However, serum copper and ceruloplasmin levels are not reliable diagnostic biomarkers due to the low concentrations in healthy newborns either. The featured imaging manifestations play an important role in diagnosing Menkes disease. To our knowledge, there are few reports on the systemic imaging manifestations of Menkes disease.

**Case presentation:**

A 4-month-old male patient presented with recurrent seizures. He had cognitive, intellectual, growth, gross motor, precision movement, and language developmental lags. The patient’s hemoglobin and serum ceruloplasmin level were low. On MRI, increased intracranial vascular tortuosity, cerebral and cerebellar atrophy, white matter changes, and basal ganglia abnormalities were observed. Plain radiograph revealed wormian bones, rib flaring, metaphyseal spurring, and periosteal reactions in the long bones of the limbs. A pathogenic variant in *ATP7A* gene was identified in the patient, so he was confirmed the diagnosis of Menkes disease. His symptoms did not improve despite symptomatic and supportive treatment during his hospitalization. Unfortunately, the infant died 3 months after leaving hospital.

**Conclusion:**

A comprehensive and intuitive understanding of the disease’s imaging manifestations can help clinicians to identify the disease and avoid delays in care.

## Background

Menkes disease (MD) is a rare X-linked genetic disorder with multisystem involvement due to copper transport defects caused by mutations in the *ATP7A* gene. This was first reported in 1962 by John H. Menkes et al. [1]. It is characterized by progressive neurological degeneration and connective tissue abnormalities. The incidence of Menkes disease is low, ranging from 1 in 100 000 to 1 in 300 000 births [2]. Affected infants often appear healthy at birth until 2–3 months of age, when they exhibit feeding difficulties, recurrent seizures, hypotonia, and delayed developmental milestones. Physical examination may reveal distinctive features, such as coarse, sparse, and lightly pigmented kinky hair; a jowly appearance with sagging cheeks; and lax skin. Tragically, without treatment, most patients die in early childhood, usually before the age of 3 [3]. Early recognition of the disease is important to facilitate early intervention and treatment. A systematic understanding of the imaging manifestations of the disease can help clinicians identify the disease in time.

### Case presentation

**Fig. 1:**
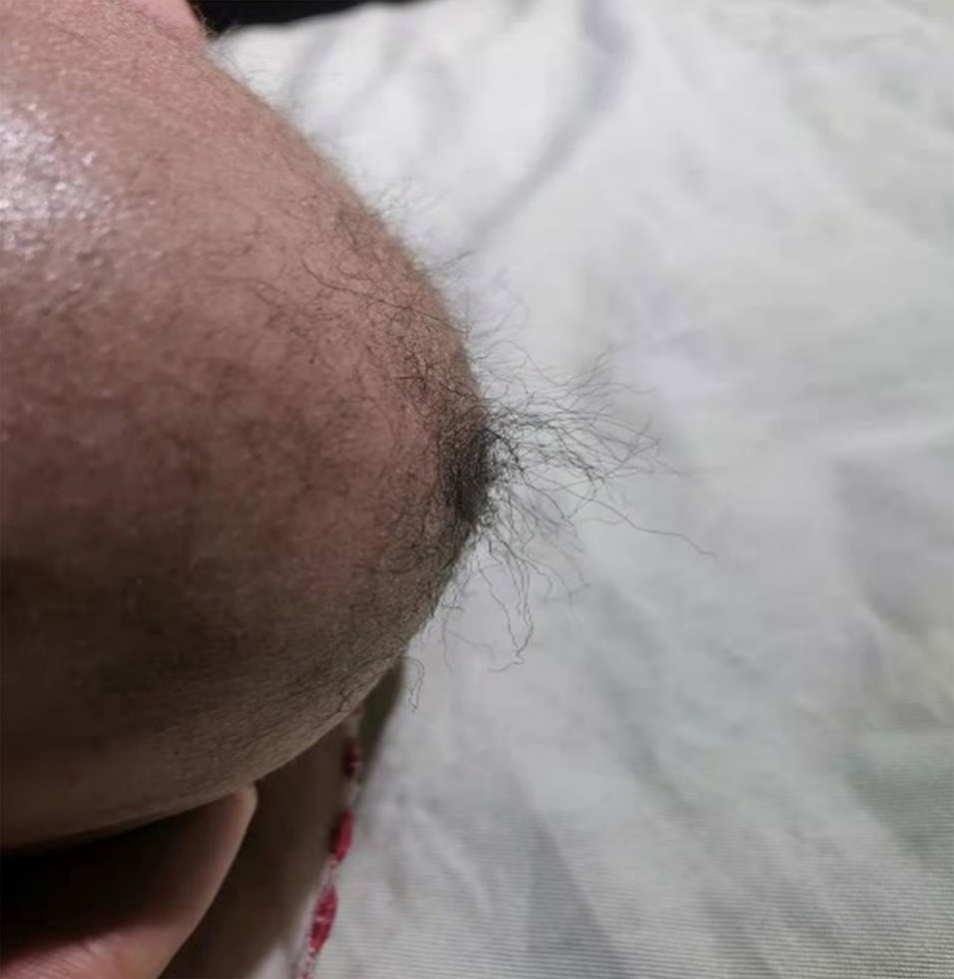
The infant has short, sparse, coarse and kinky hair on the back of the head

A 4-month-old male patient presented with recurrent seizures, with squeezing of the left eye and tilting of the corner of the mouth toward the left. Each seizure lasted approximately 10 s, with approximately 10 episodes per day that resolved independently. The infant was born prematurely at 34 weeks of gestation and presented with prolonged physiological jaundice during the neonatal period. The infant was unable to hold his head upward. He had cognitive, intellectual, growth, gross motor, precision movement, and language developmental lags. His height and weight were 0.60 m and 5.8 kg, respectively (body mass index = 16.11 kg/m^2^), which were two standard deviations lower than the average for the same age and sex. His head circumference was 41 cm, falling within one standard deviation (1.2 cm) of the peers. He had sparse, coarse, twisted, and lightly pigmented hair on the back of the head (Fig. 1) and bilateral inguinal hernias. Unfortunately, the older brother of the infant, who was diagnosed with epilepsy, died at the age of 2 years.

**Fig. 2:**
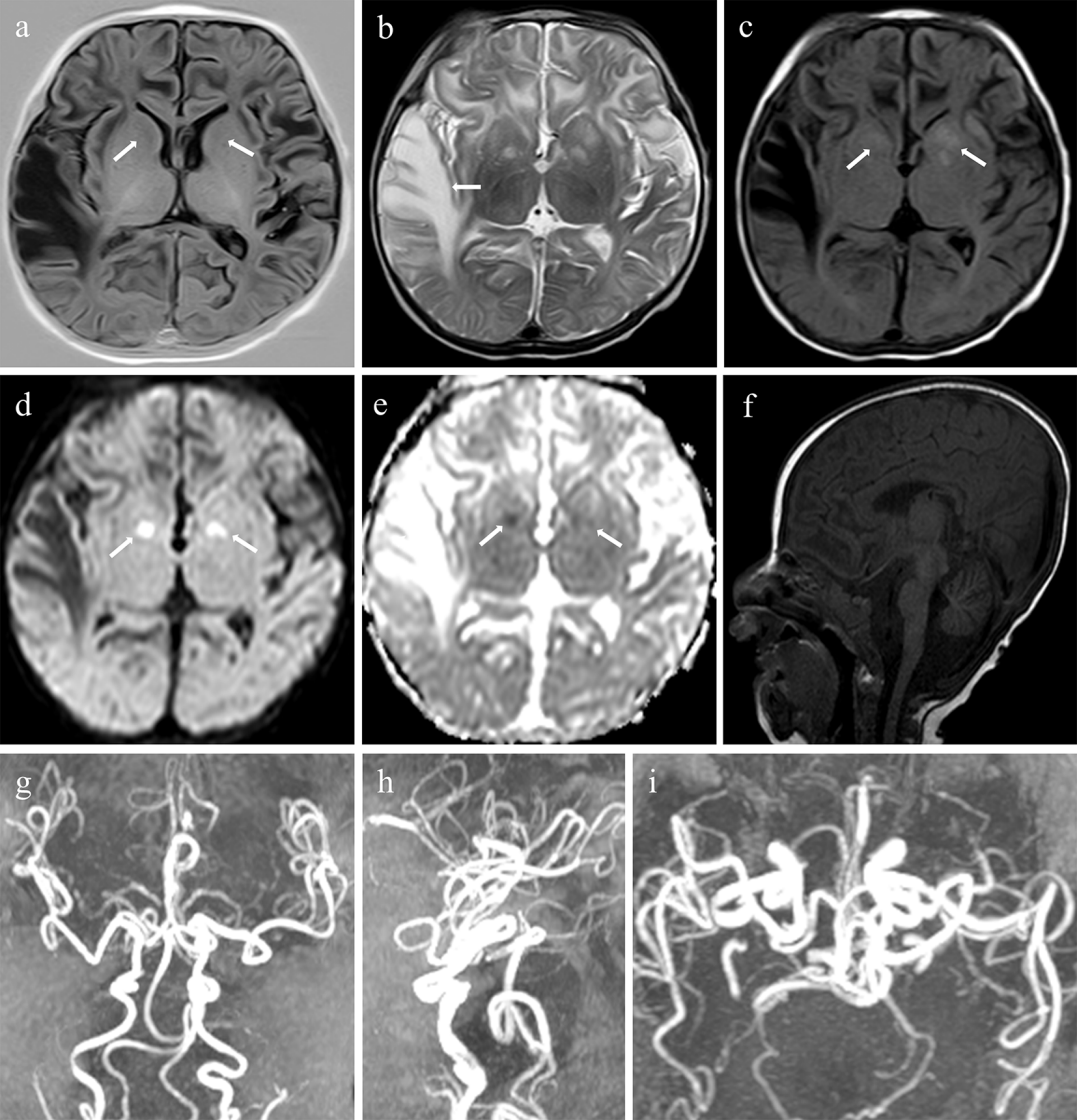
Cranial MRI images. (**a-b**) Axial T1-weighted image (T1WI) shows the delayed myelination (arrows). Axial T2-weighted image (T2WI) shows the distinctive tumefactive lesion in the right temporal lobe (arrow). (**c-e**) Axial T2-fluid-attenuated inversion recovery (T2-FLAIR) shows asymmetric hyperintensity in bilateral head of caudate nucleus and globus pallidus (arrows). Diffusion-weighted image (DWI) (b value = 800 s/mm2) and apparent diffusion coefficient (ADC) maps show asymmetric ovoid foci of restricted diffusion in bilateral globus pallidus (arrows). (**f**) Sagittal T1WI shows cerebellar atrophy. (**g-i**) Magnetic Resonance Angiography (MRA) shows increased tortuosity of the internal carotid, anterior cerebral, middle cerebral, posterior cerebral, vertebral and basilar arteries

The patient’s hemoglobin level was low at 81 g/L (normal > 90 g/L), serum ceruloplasmin level low at 37 mg/L (normal 110–620 mg/L), and serum pyruvate level high at 315 µmol/L (normal 20–100 µmol/L), serum β-hydroxybutyric acid unremarkable. A cerebrospinal fluid (CSF) test revealed a high lactic acid level of 45 U/L (normal 5–35 U/L). A cranial MRI (PHILIPS Achiva 1.5T Nova Dual) performed during hospitalization revealed multiple intracranial abnormalities: (i) The patient showed white matter changes. T1-weighted image (T1WI) revealed delayed myelination in the brain. T2-weighted image (T2WI) showed massive white matter hyperintensities in both cerebral hemispheres, with tumefactive lesions in the right temporal lobe (Fig. 2a and b). (ii) The patient showed basal ganglia abnormalities. T2-fluid-attenuated inversion recovery (T2-FLAIR) showed asymmetric hyperintensity in the bilateral heads of the caudate nucleus and globus pallidus. Diffusion-weighted and apparent diffusion coefficient maps revealed asymmetric ovoid lesions with restricted diffusion in the bilateral globus pallidus (Fig. 2c and e). (iii) The patient showed cerebral and cerebellar atrophy (Fig. 2f). (iv)The patient showed intracranial blood vessel abnormalities. Magnetic Resonance Angiography (MRA) showed an increased tortuosity of the internal carotid, anterior cerebral, middle cerebral, posterior cerebral, vertebral, and basilar arteries (Fig. 2g and i)

**Fig. 3:**
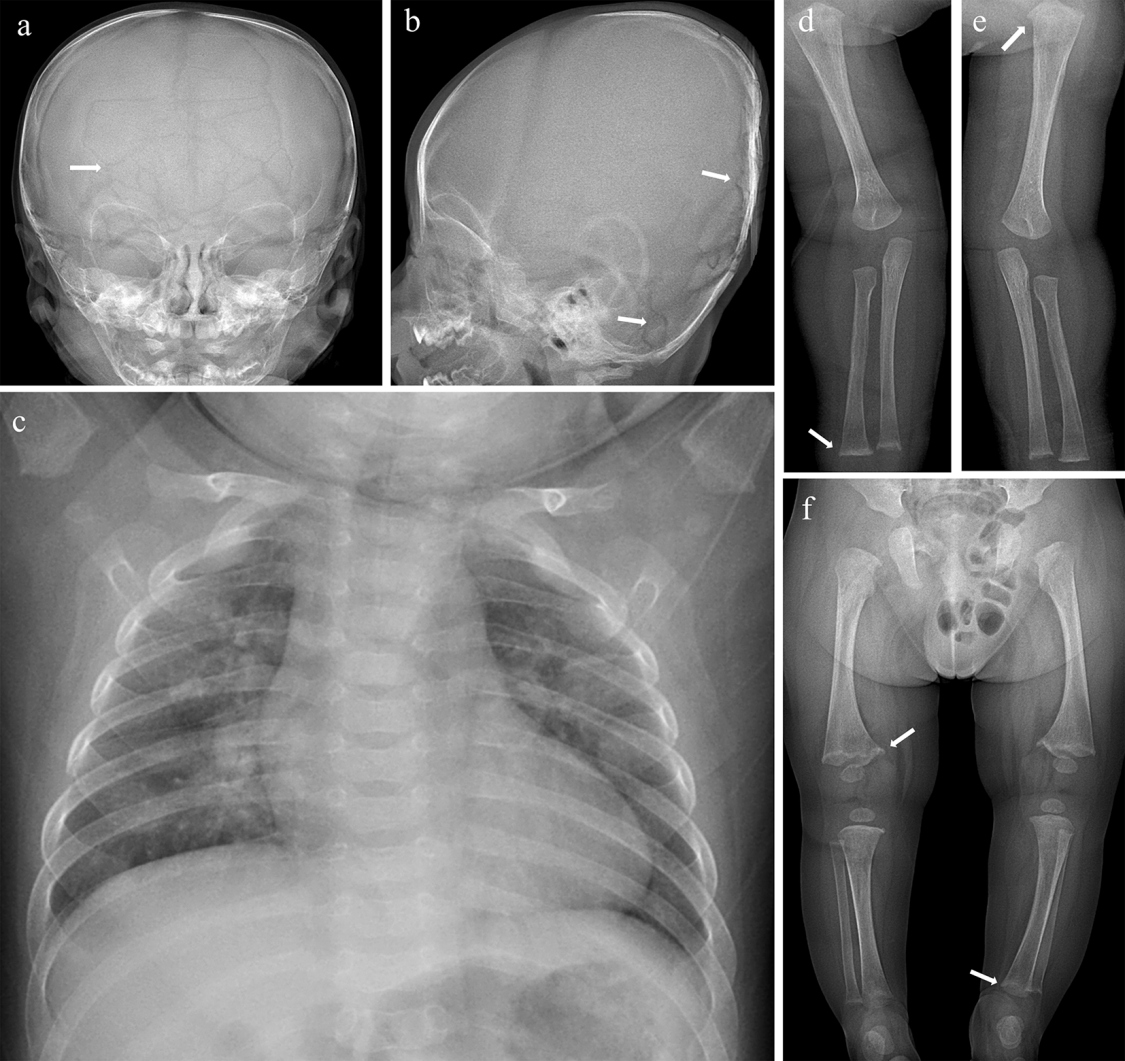
Plain radiograph images. (**a-b**) Anteroposterior and lateral skull radiograph shows numerous wormian bones along the sagittal suture, bilateral lambdoid sutures and mastoid fontanelle (arrows). (**c**) Anteroposterior chest radiograph shows flaring and expansion of anterior ends of bilateral ribs. (**d-f**) Enlargement and mineralization of metaphysis with lateral spurs involving right ulnar, left humerus, right femur and left tibia (arrows)

After discovering abnormalities in the patient’s serum and cerebrospinal fluid metabolic markers, the clinicians initially suspected a diagnosis of Menkes disease. In order to understand the overall skeletal condition, a full-body X-ray examination was conducted. Plain radiography of the entire body revealed bone abnormalities: (i) Anteroposterior and lateral skull radiographs showed numerous wormian bones in the sagittal suture, bilateral lambdoid sutures, and mastoid fontanelle (Fig. 3a and b). (ii) Anteroposterior chest radiograph showed flaring and expansion of the anterior ends of bilateral ribs (Fig. 3c). (iii) Anteroposterior upper and lower extremity radiographs showed enlargement and mineralization of the metaphysis with excessive lateral spurs involving the humerus, ulna, radius, femur, tibia, and fibula. Some mild osteopenia but no fractures were observed (Fig. 3d and f)

A variant (NM_000052.7: exon 15:c.3076delA(p.Ile1026*)) in *ATP7A* was identified in the patient and classified as pathogenic according to the American College of Medical Genetics and Genomics classification (ACMG) guideline [4]. At admission, the patient was treated with ceftriaxone and amoxycillin clavulanate potassium to prevent infection, with levetiracetam to control seizure, and with vitamins, levocarnitine to improve metabolic conditions. His symptoms have not improved and unfortunately died 3 months after leaving hospital.

## Discussion and conclusions

Menkes disease is associated with abnormal copper metabolism in the body. Copper is an essential micronutrient required for the activity of critical metabolic reactions, signaling pathways and redox reactions [5]. The copper-transporting ATPase *ATP7A* acts as an important cofactor for connective tissue and neuronal copper-dependent enzymes, such as tyrosinase, peptidyl­α­amidating monooxygenase (PAM), lysyl oxidase (LOX), dopamine-β hydroxylases (DβH), and cytochrome c oxidase (COX) [6–8]. In normal conditions, *ATP7A* enables the uptake of copper from the intestinal brush border, to be transported across cells to various tissues and organs throughout the body [9]. In Menkes disease, mutations in the *ATP7A* gene (including deletion ranging in size from a single exon to almost the entire gene, missense and splice-site mutations, exon duplications, and point mutations) may lead to impaired protein function, protein misfolding, and rapid degradation [7]. This results in the inadequate uptake of copper by the intestinal epithelium and impaired cross-cellular transport, ultimately leading to abnormal copper distribution with severe lack in blood, brain, and liver, but high values in intestines, heart, kidneys spleen, pancreas and skeletal muscle [10].

In this patient, elongation and tortuosity of the intracranial blood vessels, numerous wormian bones, flaring of the ribs, and metaphyseal changes in the long bones were observed. The pathogenic mechanism of tortuous blood vessels may be related to impaired elastin and collagen cross-linkages due to the impairment of LOX activity. Normal cross-linking of collagen is thought to be essential for the mineralization of bone tissue [9]. Cerebral and cerebellar atrophy is another typical feature that likely contributes to the combined effects of metabolic impairment, prolonged status epilepticus, lactic acidosis, and mitochondrial failure [11–13]. White matter abnormalities vary among individuals, the lesions show vasogenic edema-like features. However, the exact pathogenic mechanism is uncertain and may be related to altered blood-brain barriers due to impaired copper metabolism [14]. Typically, the bilateral but asymmetric involvement of the basal ganglia results in cytotoxic edema, which may contribute to mitochondrial dysfunction in the respiratory chain [15].

In addition to these common imaging presentations, Manara et al. [15] reported that subdural collections could be detected on MRI. Matthias K. Bernhard et al. [16] reported a 4-month-old child demonstrated dural sinus widening on cerebral angiography. Grange et al. [17] reported a case showed aneurysmal dilatation of the right internal jugular vein, along with dilatation of the superior vena cava, the odd vein, and the innominate vein on magnetic resonance venography (MRV). The chest computed tomography (CT) and computer tomography pulmonary angiography (CTPA) revealed panlobular emphysema and multiple pulmonary alveoli in both lungs, along with pulmonary hypoplasia and tortuous dilatation of the pulmonary arteries. The autopsy findings of this child revealed multiple polyps in the stomach and sigmoid colon. Kim and Zaffanello et al. [18, 19] reported that the most common complicating urologic abnormality in MD is multiple diverticula of the bladder, the others include bladder stones, neurogenic bladder, vesicoureteral reflux, ureteral effusion, renal scarring and cryptorchidism. Peng et al. [20] reported a 4-year-old boy presented with a spontaneous retroperitoneal hematoma. Osteoporosis and fracture of the long bones of the extremities without clear history of accidental trauma have been reported [21, 22]. Hill et al. [23] reported a clinical trial that included 35 children with Menkes’ disease and found a combined C2 posterior arch defect in approximately 11%.

The differential diagnosis of MD can vary among different body systems according to imaging features. Wilson’s disease, another copper metabolism disorder, may be considered in cases involving the basal ganglia, thalamus, and brainstem [24]. However, it typically exhibits less extensive white matter involvement and cerebral and intracranial arterial tortuosity. The other copper metabolism diseases, such as Huppke-Brendel Syndrome, MEDNIK syndrome and CCS deficiency, should be considered. Huppke-Brendel Syndrome presents with hypomyelination, cerebellar hypoplasia, and wide subarachnoid spaces, without basal ganglia, intracranial vessels and extensive white matter involvement. Moreover, the wormian bones have not been reported. MEDNIK syndrome (mental retardation, enteropathy, deafness, peripheral neuropathy, ichthyosis, keratodermia) mainly shows cerebral atrophy and bilateral symmetrical hyperintensities in caudate nuclei and putamina at T2-weighted images and severe osteoporosis on X-ray. These are similar to that seen in Wilson’s disease. CCS deficiency displays progressive brain atrophy, hypomyelination and symmetrical lesions of the thalami, but absence of vascular abnormalities. Occipital Horn Syndrome, known as the milder form of MD, has similar hair and connective tissue abnormalities. The main distinction is radiographic finding of the peculiar occipital horns, which are symmetric exostoses extend from the occipital bone [25–27]. Moyamoya disease (MMD), characterized by progressive stenosis, occlusion, and twisted vascular anastomoses in the circle of Willis, is a potential differential diagnosis in children with excessive tortuosity of intracranial vessels, but it differs from Menkes disease in terms of the pattern of vascular abnormalities [28]. Metabolic bone diseases, such as rickets, scurvy, and osteogenesis imperfecta, can be considered; however, numerous wormian bones and progressive neurodegenerative changes are not common features in these disorders.

There is currently no curative treatment for the patients of Menkes disease. Symptomatic and supportive measures remain the mainstay management strategies, such as seizure management, feeding or gastrostomy tube placement to enhance caloric intake, antibiotic prophylaxis to prevent infection and surgery for bladder diverticula [3, 8]. The researchers suggest that subcutaneous copper histidine injections at early age (usually in the neonatal period) have been shown effectively to improve neurodevelopment outcomes, decrease seizures frequency and severity, and increase survival [29, 30]. Recently, adeno-associated viral gene therapy in combination with copper and a membrane traversing drug like elesclomol or elesclomol-Cu^2+^ hold promise for the treatment [31, 32].

Even though the typical physical examination signs of the patients are distinctive, early diagnosis might be difficult for clinicians due to the rarity. The increased ratios of dopamine to norepinephrine, as well as dihydroxyphenylacetic acid to dihydroxyphenylglycol, are a promising test for neonatal diagnosis of Menkes disease [3]. Meanwhile, radiologists can provide more diagnostic ideas based on the featured imaging findings, such as numerous wormian bones, cerebral and cerebellar atrophy, tortuous intracerebral vessels with parenchymal abnormalities. Due to the multisystem involvement of the disease, differential diagnosis might be difficult for radiologists. Systemic and comprehensive evaluation of radiological findings, detailed physical examination and biochemical testing are important for early diagnosis.

## Data Availability

No datasets were generated or analysed during the current study.

## Abbreviations

MD: Menkes Disease
MRI: Magnetic Resonance Imaging
MRA: Magnetic Resonance Angiography
CSF: Cerebrospinal fluid
T1WI: T1-weighted image
T2WI: T2-weighted image
T2-FLAIR: T2-fluid-attenuated inversion recovery
ACMG: American College of Medical Genetics and Genomics classification
PAM: Peptidyl­α­amidating monooxygenase
LOX: Lysyl oxidase
DβH: Dopamine-β hydroxylases
COX: Cytochrome c oxidase
MRV: Magnetic resonance venography
CT: Computer tomography
CTPA: Computer tomography pulmonary angiography
MMD: Moyamoya disease
